# Supplementary material for: Beaver dams overshadow climate extremes in controlling riparian hydrology and water quality
Source: Nat Commun. 2022 Nov 8;13:6509. doi: 10.1038/s41467-022-34022-0 (PMC9643325; doi:10.1038/s41467-022-34022-0)
Supplement: Supplementary file 1 — Supplementary Information [file 41467_2022_34022_MOESM1_ESM.pdf]

Beaver dams overshadow climate extremes in controlling riparian hydrology and water quality

Supplementary Information

Christian Dewey[1], Patricia M. Fox[2], Nicholas J. Bouskill[2], Dipankar Dwivedi[2], Peter Nico[2], Scott Fendorf[1*]

[1]Earth System Science Department, Stanford University, Via Ortega 473, Stanford, CA 94305, USA
[2]Earth and Environmental Sciences Area, Lawrence Berkeley National Laboratory, Berkeley, CA, 94720, USA

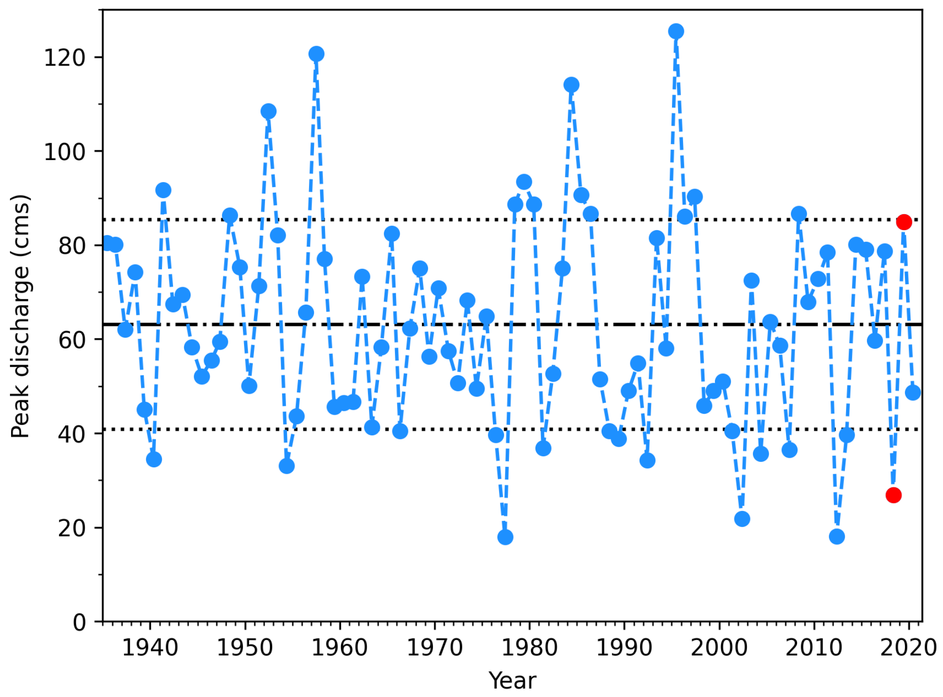

Supplementary Figure 1. **Peak discharge in the East River, as measured at the USGS stream gauge in Almont, Colorado, U.S.A. from 1935-2021**. The red markers indicate the years in which this study was conducted (2018 and 2019). The black dashed-dotted line indicates the mean peak discharge, while the dotted lines indicate standard deviation (+/-) from the mean.

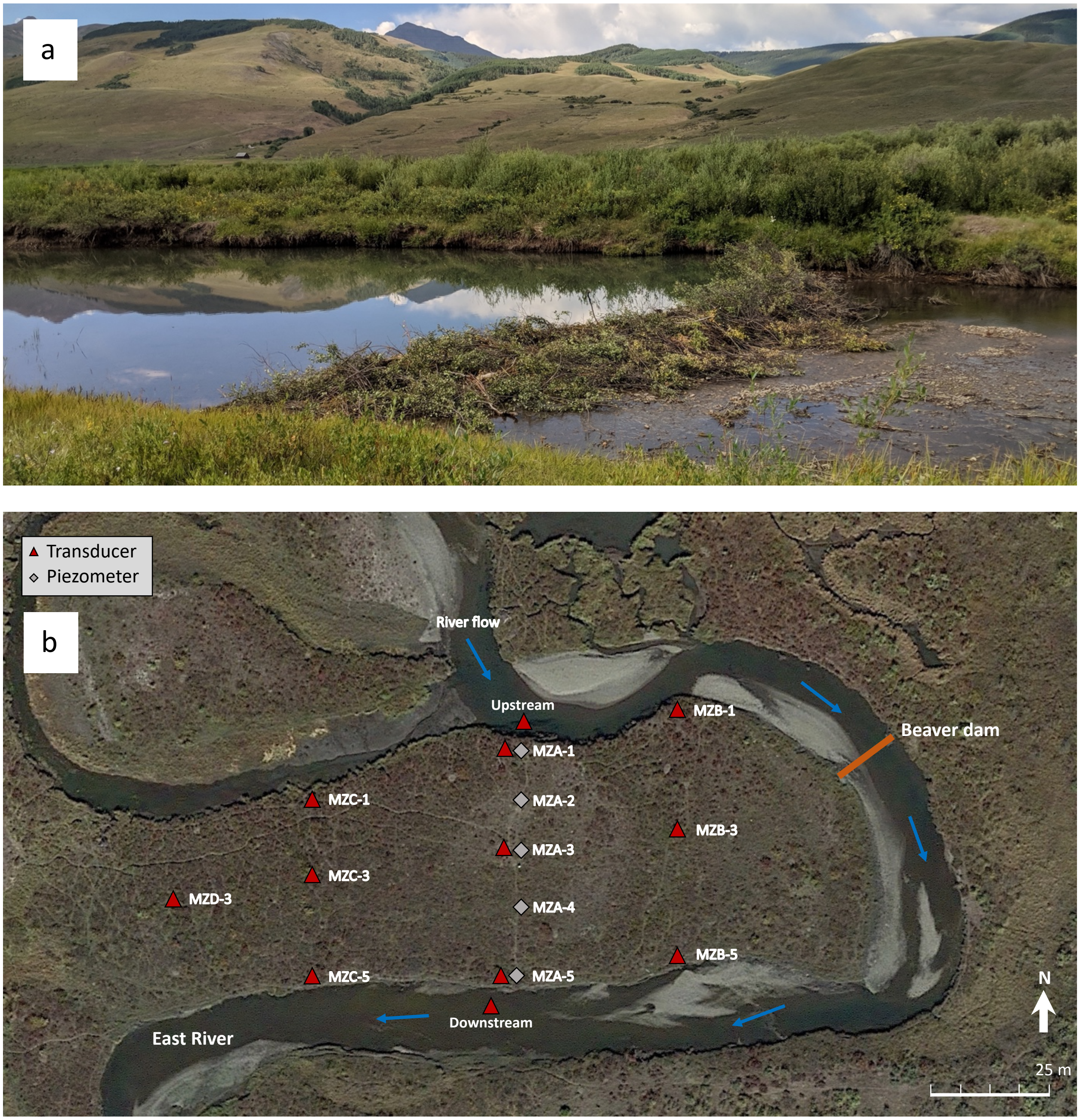

Supplementary Figure 2. **Picture of beaver dam and site map. a** The beaver dam at Meander Z on August 11, 2018. Construction of the dam began on July 29, 2018. Photo by Christian Dewey. **b** Map derived from Google Earth^{TM} satellite imagery (Landsat/Copernicus, seen on October 2, 2019) showing the location of the dam relative to the riparian zone and field instrumentation at the site. Transducers measured hourly pressure conditions, while the piezometers were used for groundwater sample collection.

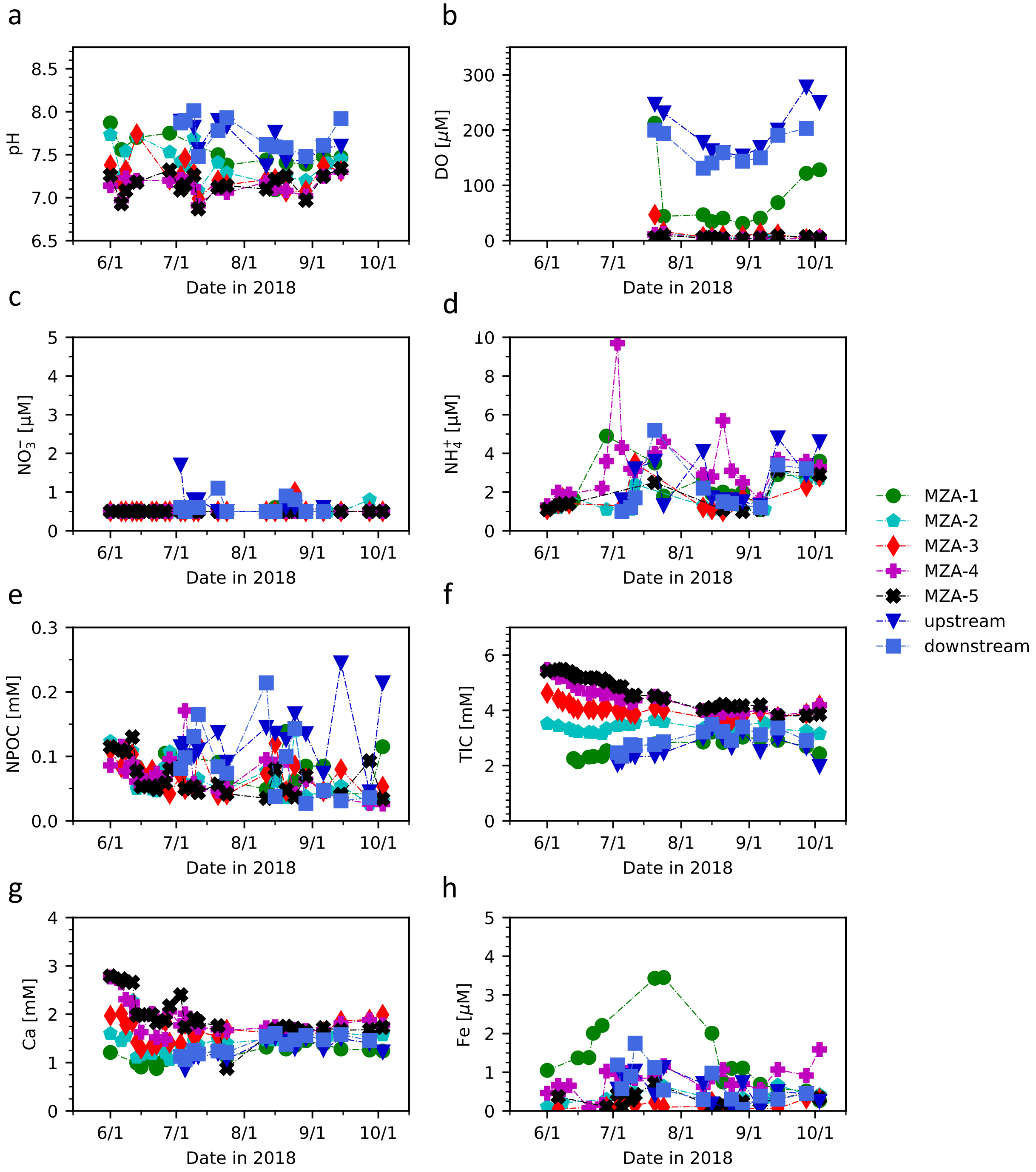

Supplementary Figure 3. **Porewater chemistry along the MZA transect in 2018.** Porewater pH (**a**), dissolved oxygen (**b**), nitrate (**c**), ammonium (**d**), non-purgeable dissolved organic carbon (NPOC) **(e)**, total inorganic carbon (TIC) **(f)**, calcium (**g**), and total dissolved iron (Fe) (**h**) along the MZA transect in 2018. Green circles represent MZA-1; turquoise pentagons represent MZA-2; red diamonds represent MZA-3; purple crosses represent MZA-4; black rotated crosses represent MZA-5; dark blue triangles and light blue squares represent the upstream and downstream reaches of the East River, respectively.

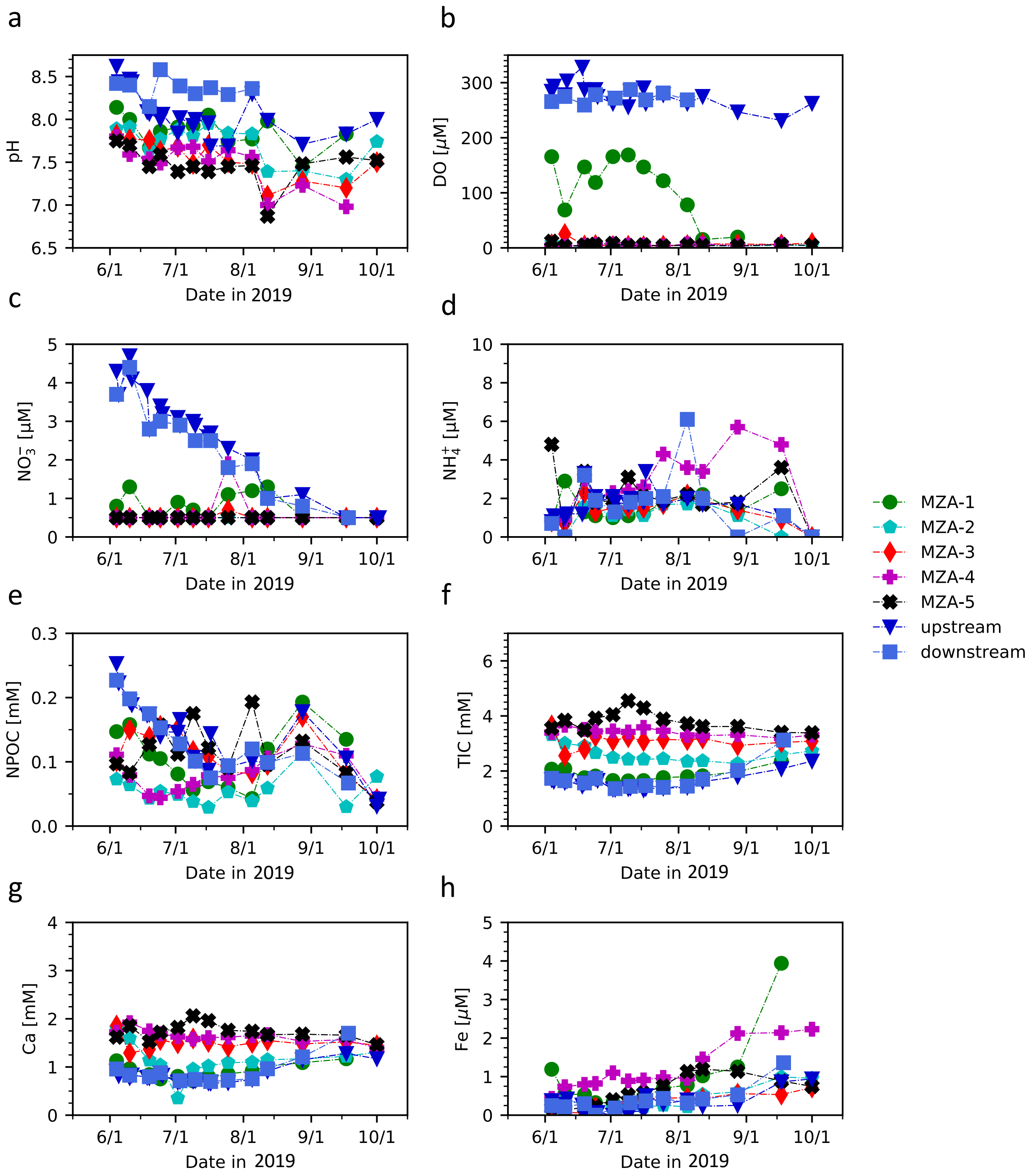

Supplementary Figure 4. **Porewater chemistry along the MZA transect in 2019.** Porewater pH (**a**), dissolved oxygen (**b**), nitrate (**c**), ammonium (**d**), non-purgeable dissolved organic carbon (NPOC) **(e)**, total inorganic carbon (TIC) (**f**), calcium (**g**), and total dissolved iron (Fe) (**h**) along the MZA transect in 2019. Green circles represent MZA-1; turquoise pentagons represent MZA-2; red diamonds represent MZA-3; purple crosses represent MZA-4; black rotated crosses represent MZA-5; dark blue triangles and light blue squares represent the upstream and downstream reaches of the East River, respectively.

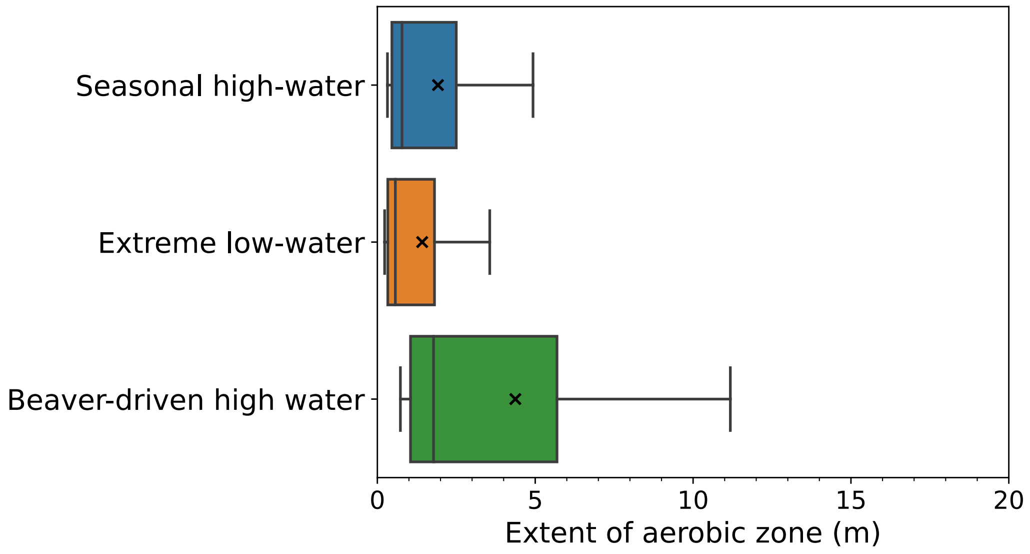

Supplementary Figure 5. **Results of Monte Carlo analysis of extent of aerobic zone.** Distribution of distances to Da_{DO} = 1 at seasonal high-water conditions (top), extreme low-water conditions (middle), and beaver-driven high-water conditions (bottom) across the range or possible aerobic respiration and nitrification rates. Quantiles of the distributions are represented in the box and whisker plots. The cross symbols indicate the distance to Da_{DO} as determined in the base simulation.

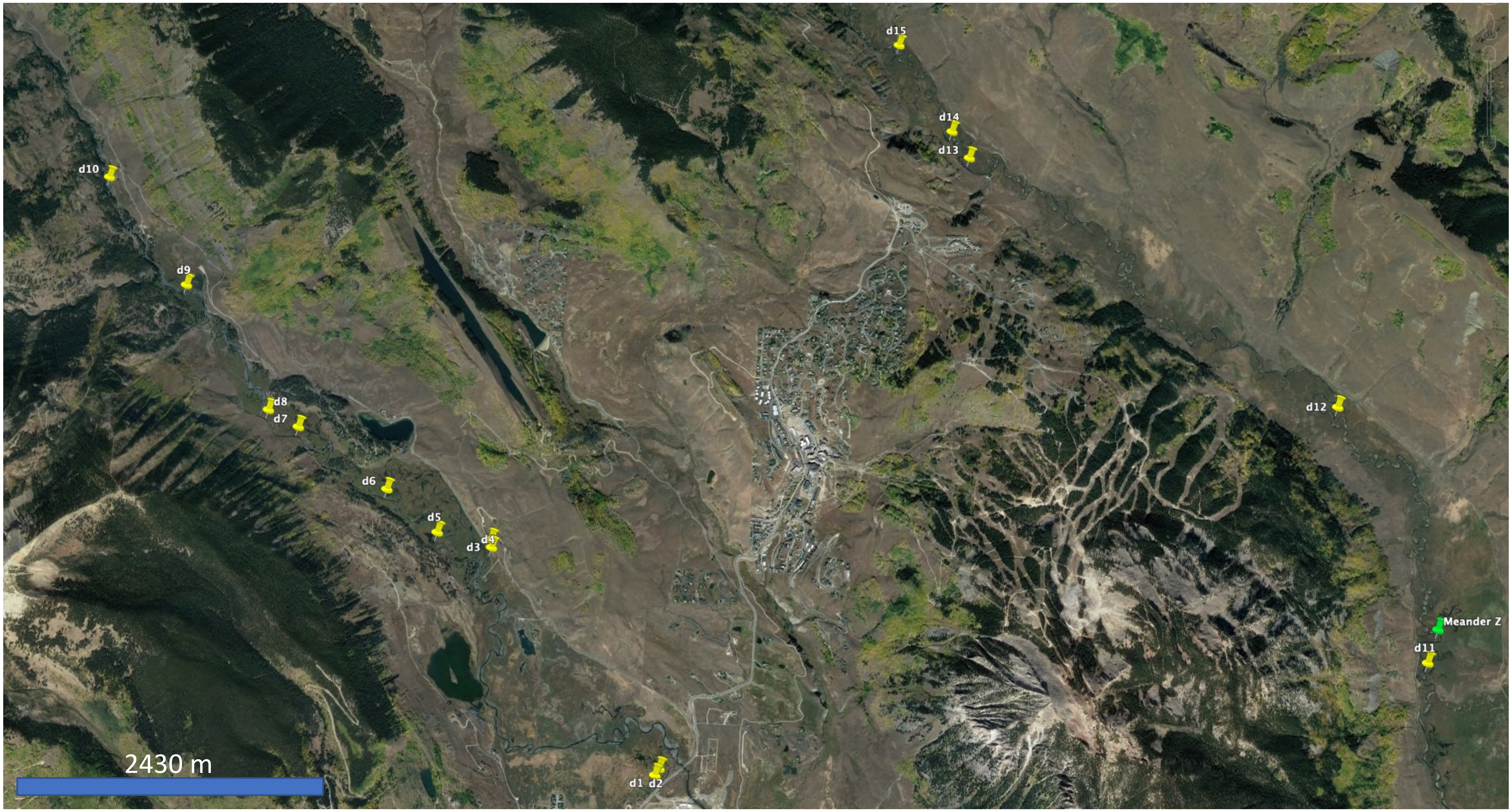

Supplementary Figure 6**. Beaver dams unambiguously identified in the East River watershed in Google Earth™ satellite imagery (Landsat/Copernicus, seen on October 2, 2019)**. Images of each individual dams are shown in Supplementary Figure 7.

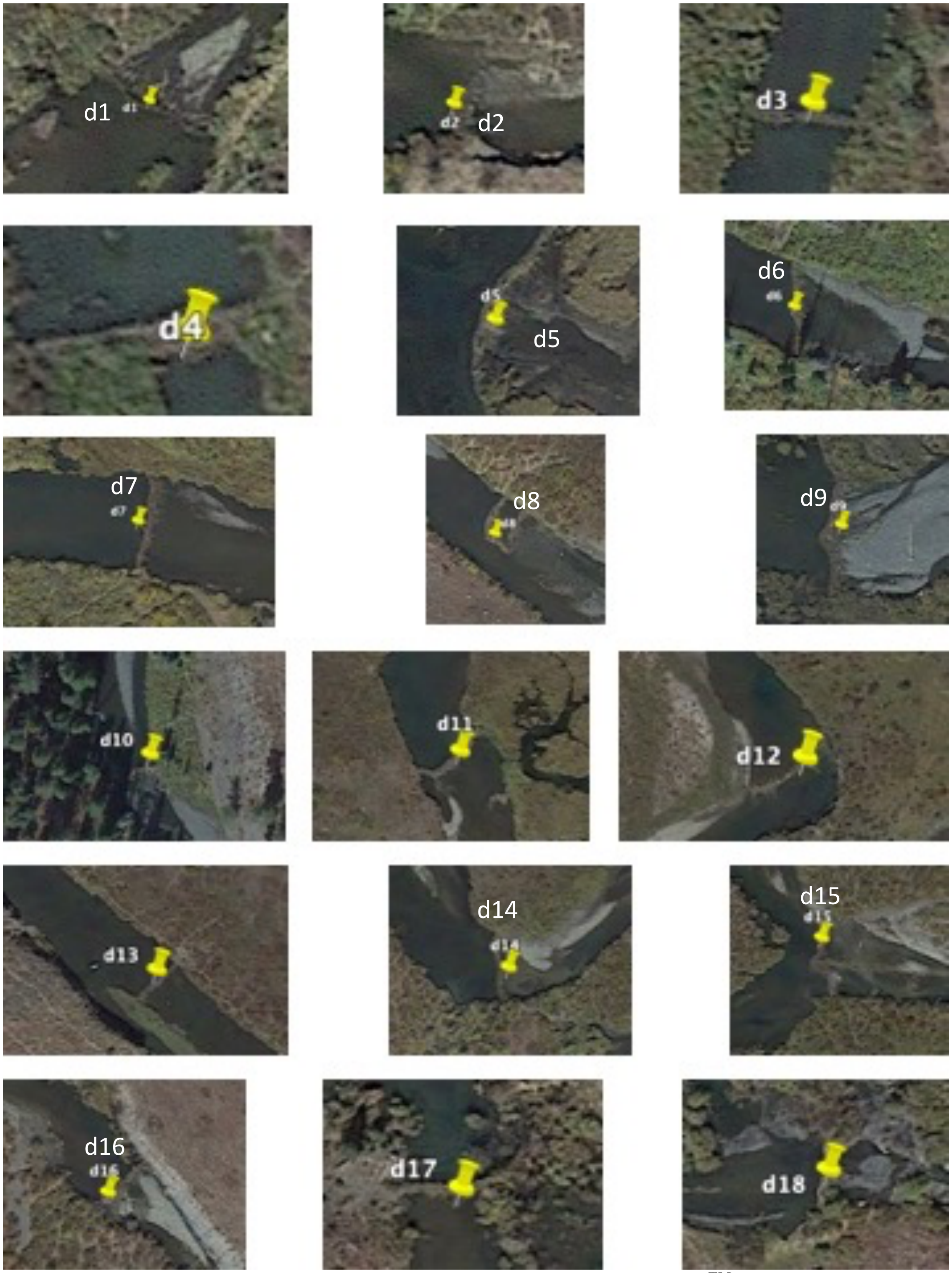

Supplementary Figure 7. **Individual beaver dams identified in Google Earth™ satellite imagery (Landsat/Copernicus, seen on October 2, 2019).** Labels correspond to the labels in Supplementary Figure 6.

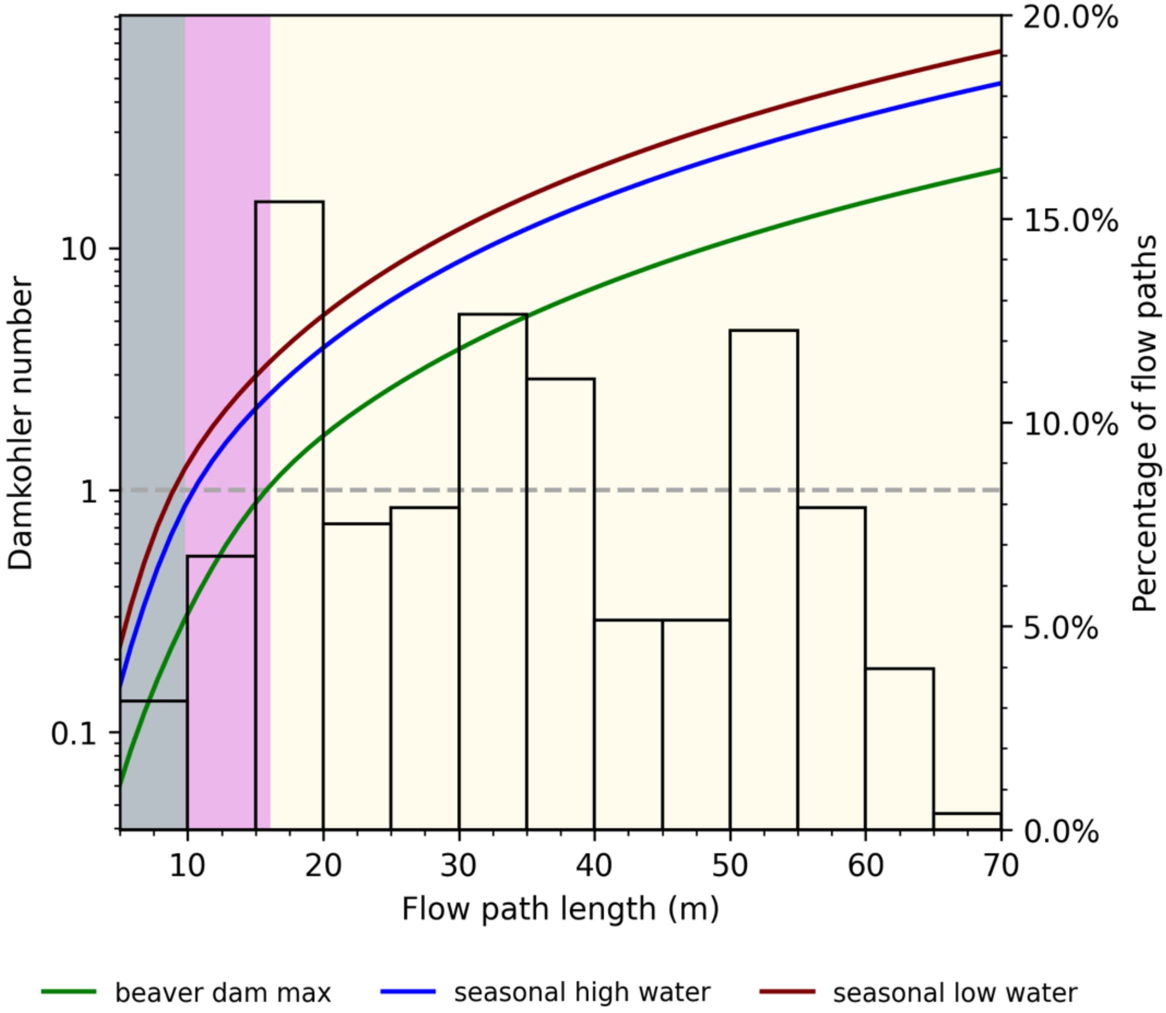

Supplementary Figure 8. **Flow paths measured within representative meandering portions of the East River floodplain**. The distribution of flow path lengths is represented in the histogram. For flow paths between 5 and 70 m, the Damköhler number relating nitrate transport and oxygen consumption was determined for the hydrologic conditions corresponding to the beaver dam maximum (green), seasonal high water (blue), and base flow conditions (maroon). A Damköhler number less than 1 indicates that transport processes control nitrate fluxes, while a Damköhler number greater than 1 indicates that the rate of oxygen consumption controls nitrate fluxes. Flow paths shaded in gray are those along which nitrate fluxes are controlled by transport processes during the three hydrologic extremes; flow paths shaded in magenta are those along which nitrate fluxes change from reaction-controlled to transport-controlled at the beaver dam maximum; and flow paths shaded in beige are those along which nitrate fluxes are controlled by reaction rates during the three hydrologic extremes.

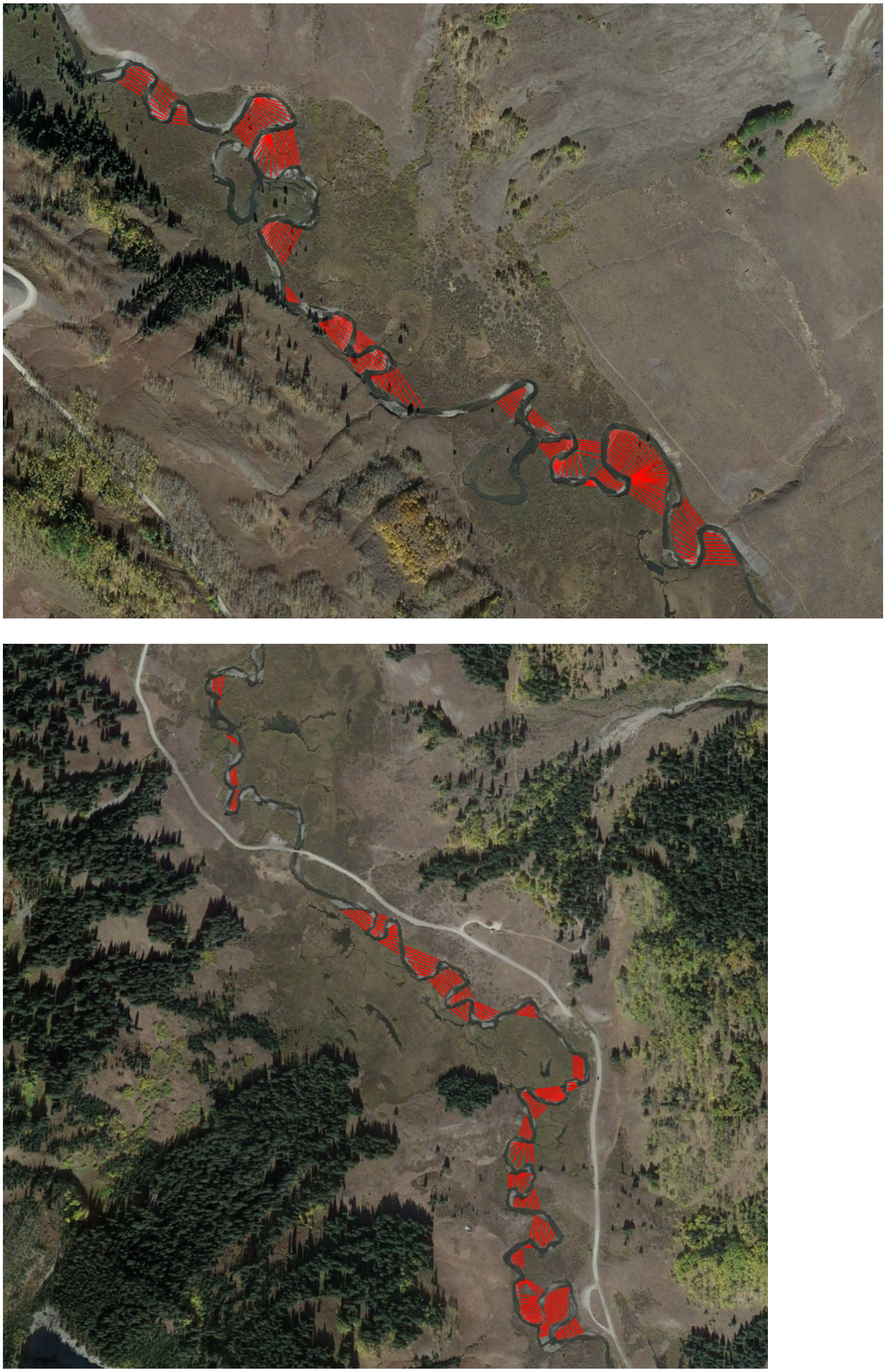

Supplementary Figure 9. **Flow paths used to determine flow path length distribution**. A total of 253 flow paths were counted using Google Earth^{TM} satellite imagery (Landsat/Copernicus, seen on October 2, 2019).

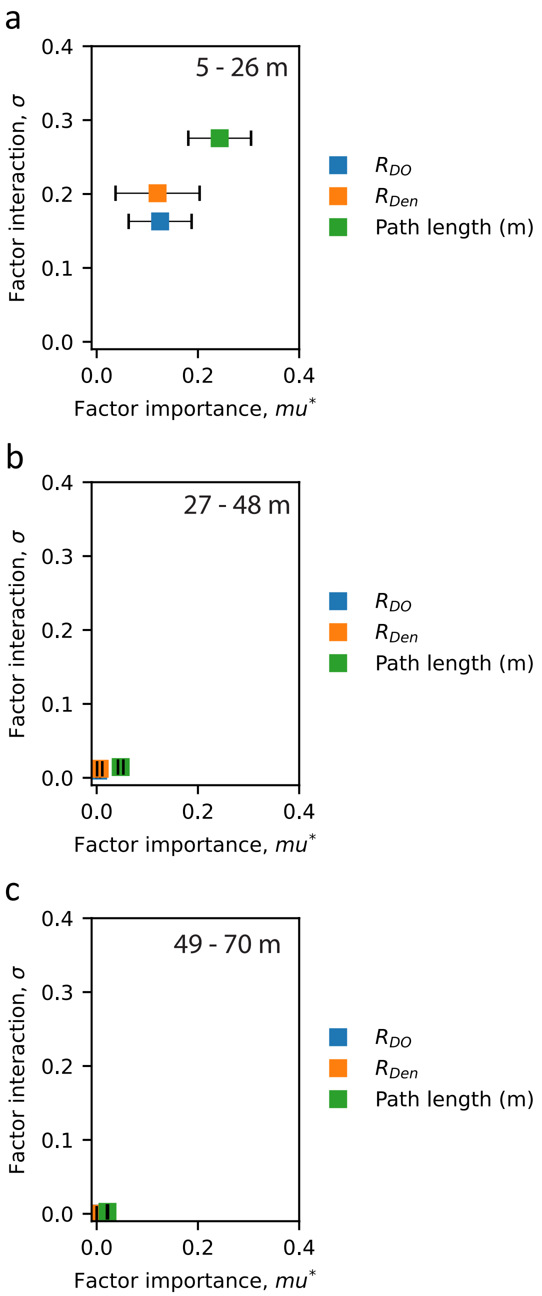

Supplementary Figure 10. **Results of the Morris sensitivity analysis for the hyporheic nitrate mass balance.** The mass balance was defined as the difference between total nitrate inflow and total nitrate outflow between April 7 and October 31, 2018 (hydrologic conditions include the beaver dam). Sensitivity was assessed across the range of possible denitrification and oxygen consumption rates (R_{Den} and R_{DO}, respectively) over three ranges of flow path lengths: 5 to 26 m (**a**); 27 to 48 m (**b**); and 49 to 70 m (**c**). A higher value of factor importance (X-axis) indicates a greater impact of the parameter on nitrate mass balance. A higher value of factor interaction (Y-axis) indicates greater interaction of the parameter with other parameters. Error bars indicate 95% confidence interval of factor effect and are predominantly a function of the path length range. In the bottom panel, the effects of R_{DO} and R_{Den} are both 0 and overlap in the plot.

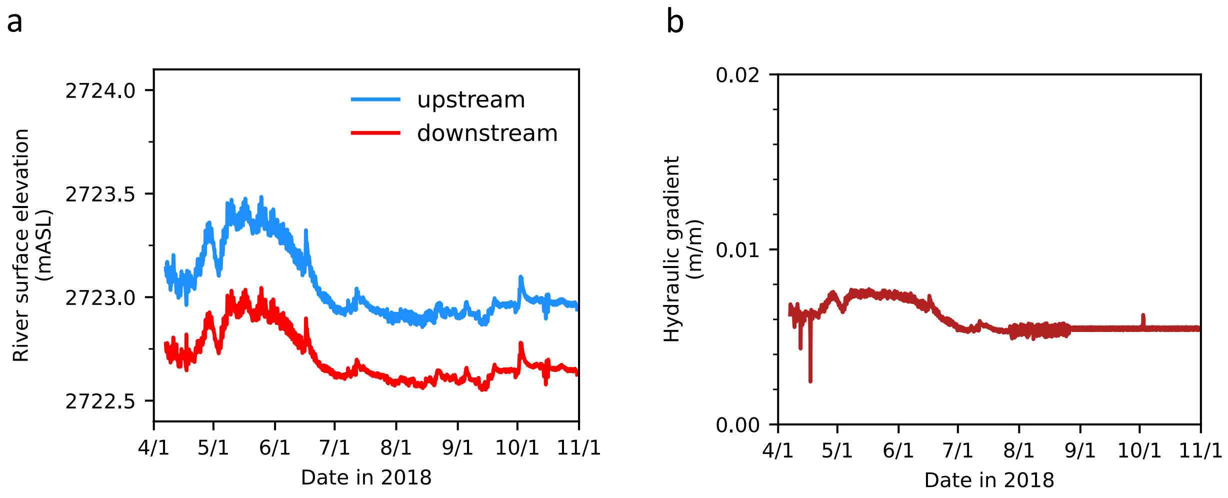

Supplementary Figure 11. **River surface elevations in 2018 with the beaver dam artificially removed. a** River surface elevations in 2018 at the upstream (blue) and downstream (red) locations, with the effects of the beaver dam artificially removed. **b** The corresponding hydraulic gradient.

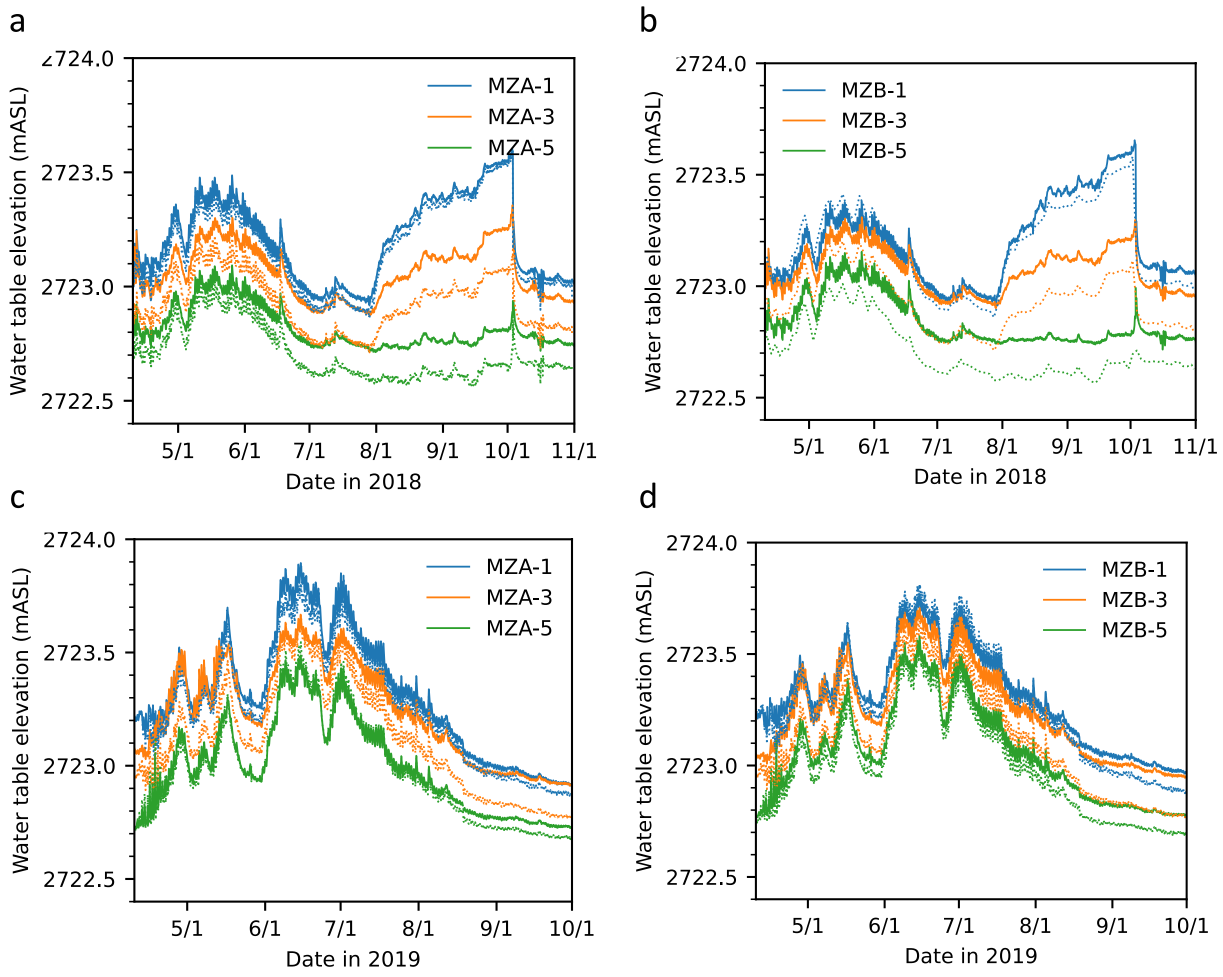

Supplementary Figure 12. **Validation of hydrologic model.** Modeled and observed water table elevations (dotted and solid lines, respectively) along the MZA (**a, c**) and MZB (**b, d**) transects in 2018 and 2019. Transducers were installed at locations 1 (blue), 3 (orange), and 5 (green) along each transect.

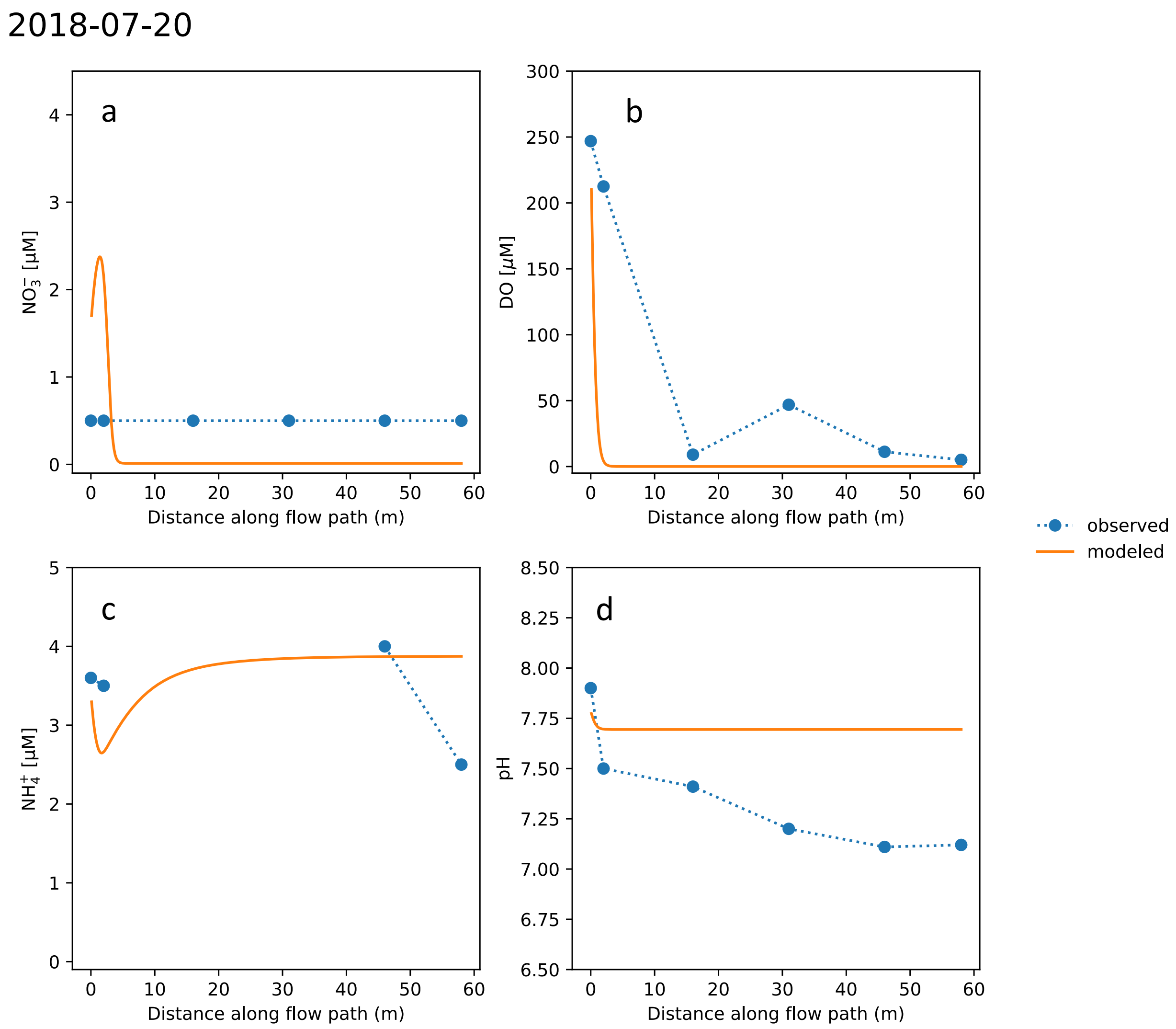

Supplementary Figure 13. **Validation of reaction network on July 20, 2018**. Observed and modeled porewater concentrations of nitrate (**a**), dissolved oxygen (**b**), and ammonium (**c**) and pH (**d**) for July 20, 2018. This date corresponds to base flow (low water) conditions at the site in 2018.

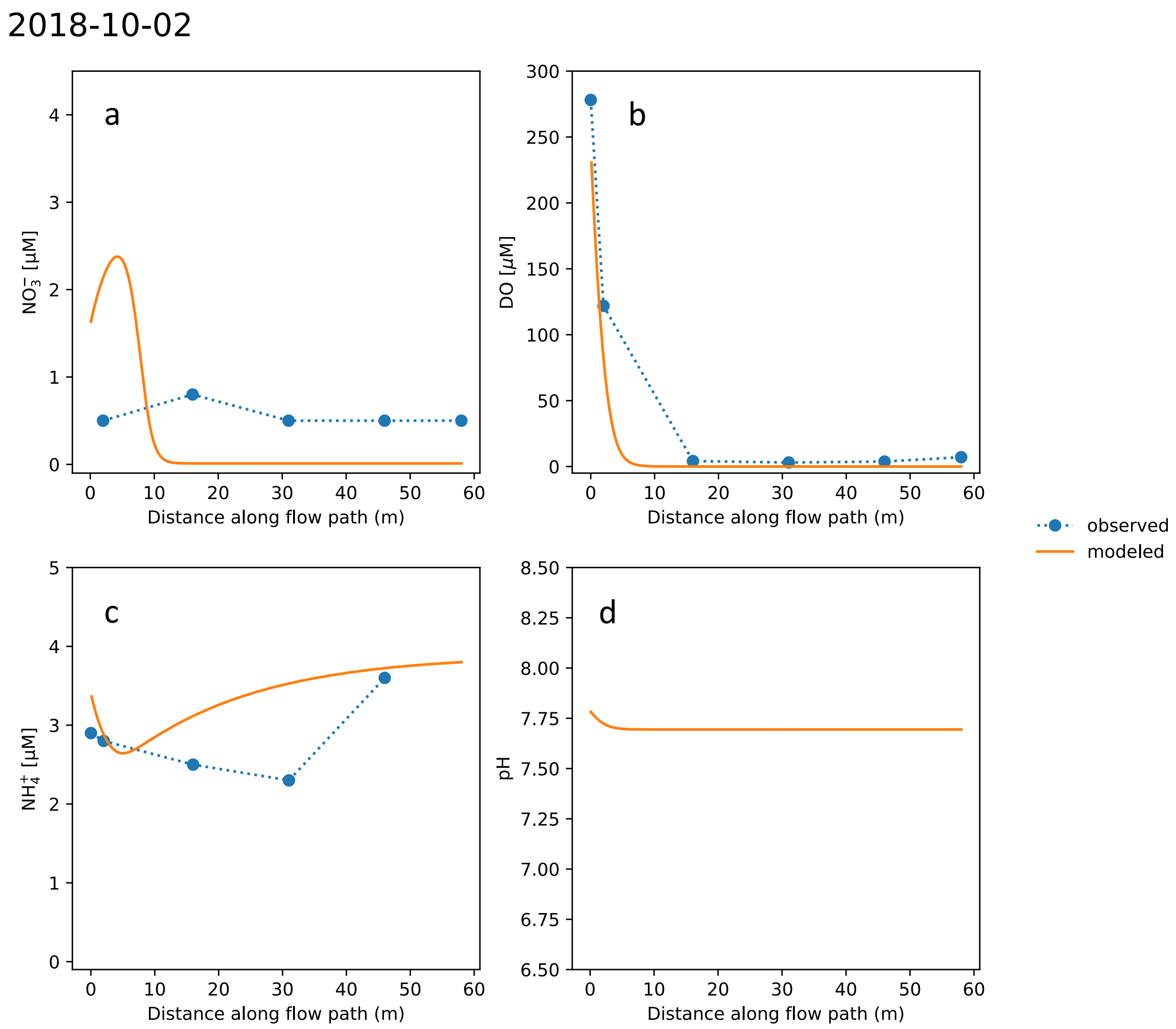

Supplementary Figure 14. **Validation of reaction network on October 2, 2018**. Observed and modeled porewater concentrations of nitrate (**a**), dissolved oxygen (**b**), and ammonium (**c**) and pH (**d**) for October 2, 2018. This date corresponds to the beaver dam maximum at the site in 2018.

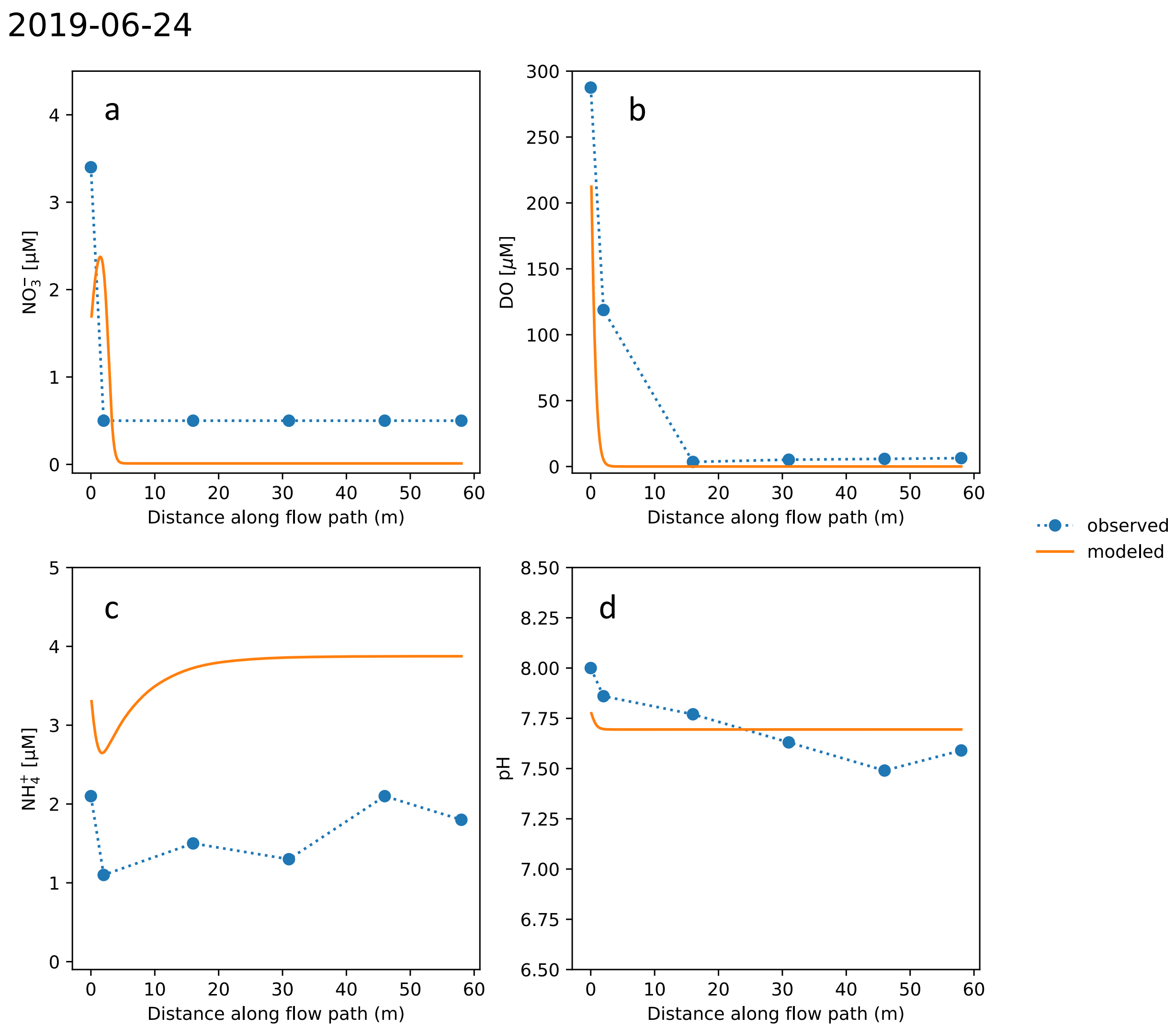

Supplementary Figure 15. **Validation of reaction network on June 24, 2019**. Observed and modeled porewater concentrations of nitrate (**a**), dissolved oxygen (**b**), and ammonium (**c**) and pH (**d**) for June 24, 2019. This date corresponds to peak water levels at the site in 2019.

TABLES

Supplementary Table 1. **Microbial reaction stoichiometry and thermodynamics**.

Reaction	Stoichiometric Reaction Equation	ΔG_{rxn} (kJ / mol C)[a]
Aerobic Resipration	0.5 CH_3COO- + 1.0 $O_{2(aq)}$ ⟶ 1.0 HCO_3- + 0.5 H+	-501
Nitrification	0.5 $NH_{3(aq)}$ + 1.0 $O_{2(aq)}$ ⟶ 0.5 NO_3- + 0.5 H_2O + 0.5 H+	-181
Denitrification	0.5 CH_3COO- + 0.8 NO3- + 0.3 H+ ⟶ 1.0 HCO_3- + 0.4 $N_{2(aq)}$ + 0.4 H_2O	-476

[a]Assumes 25 °C, pH 7

Supplementary Table 2. **Microbial reaction parameters.**

Reaction	Parameter	Value	Units	Source
Aerobic Respiration	μ_{max}	4.44×10^{-4}	(mol m^3_{bulk}) / (L mol_{bio} s)	a
	X_{max}	1.00×10^{-5}	mol_{bio} / m^3_{bulk}	b
	C_S	[Ac-]	mol / L	NA
	K_S	1.00×10^{-6}	mol / L	c
	C_{TEA}	[$O_{2(aq)}$]	mol / L	NA
	K_{TEA}	2.41×10^{-4}	mol / L	d
Nitrification	μ_{max}	1.60×10^{-4}	(mol m^3_{bulk}) / (L mol_{bio} s)	a
	X_{max}	1.00×10^{-5}	mol_{bio} / m^3_{bulk}	b
	C_S	[$NH_{3(aq)}$]	mol / L	NA
	K_S	1.48×10^{-5}	mol / L	d
	C_{TEA}	[$O_{2(aq)}$]	mol / L	NA
	K_{TEA}	2.41×10^{-4}	mol / L	d
Denitrification	μ_{max}	3.50×10^{-4}	(mol m^3_{bulk}) / (L mol_{bio} s)	d
	X_{max}	1.00×10^{-5}	mol_{bio} / m^3_{bulk}	b
	C_S	[Ac-]	mol / L	NA
	K_S	1.00×10^{-6}	mol / L	c
	C_{TEA}	[NO_3-]	mol / L	NA
	K_{TEA}	1.00×10^{-4}	mol / L	d
	C_I	[$O_{2(aq)}$]	mol / L	NA
	K_I	5.00×10^{-7}	mol / L	b

[a]fit to observations; [b]tuned to yield a reaction rate consistent with published values; [c]set with the assumption that DOC utilization was not rate-limiting at typical porewater DOC concentrations, as in Dwivedi et al.[1] and Maggi et al.[2]; [d]from Maggi et al.[2]

Supplementary Table 3. **Solid phase dissolution and organic matter mineralization reaction parameters.**

Solid Phase	Parameter	Value	Units	Source
Calcite	A_m	6.00×10^{1}	cm^2 / cm^3	a
	k_n	1.55×10^{-6}	$mol / (m^2 \cdot s)$	a
	k_{H+}	5.01×10^{-1}	$mol / (m^2 \cdot s)$	a
	k_{HCO3-}	3.31×10^{-4}	$mol / (m^2 \cdot s)$	a
	$\log k_{eq}$	1.8487	NA	a,b
SOM	A_m	1.13×10^{5}	cm^2 / cm^3	c
	k_n	1.00×10^{-5}	$mol / (m^2 \cdot s)$	d
	$\log k_{eq}$	-32.6000	NA	e
N-SOM	A_m	1.13×10^{5}	cm^2 / cm^3	c
	k_n	7.00×10^{-17}	$mol / (m^2 \cdot s)$	f
	$\log k_{eq}$	-7.0000	NA	f

[a]Palandri and Kharaka[3]; [b]Shock et al.[4]; [c]Dwivedi et al.[1]; [d]set to yield a pseudo-equilibrium reaction; [e]tuned to yield acetate concentrations consistent with field observations; [f]tuned to yield ammonium concentrations consistent with field observations.

Supplementary Table 4. **Average river water composition used for upstream and downstream chemical boundary conditions.**

Average River Water Composition		
pH	7.8	
Cl-	4.00×10^{-5}	M
SO_4^{2-}	3.50×10^{-5}	M
HCO_3-	1.50×10^{-3}	M
Ca^{2+}	1.20×10^{-3}	M
Mg^{2+}	2.18×10^{-4}	M
K^+	1.51×10^{-5}	M
Na^+	5.00×10^{-5}	M
$O_{2(aq)}$	2.41×10^{-4}	M
NO_3-	1.60×10^{-6}	M
DOC	8.00×10^{-4}	M
$NH_{3(aq)}$	3.40×10^{-6}	M

Supplementary References

1. Dwivedi, D. *et al.* Geochemical Exports to River From the Intrameander Hyporheic Zone Under Transient Hydrologic Conditions: East River Mountainous Watershed, Colorado. *Water Resour Res* **54**, 8456–8477 (2018).
2. Maggi, F. *et al.* A mechanistic treatment of the dominant soil nitrogen cycling processes: Model development, testing, and application. *J Geophys Res Biogeosci* **113**, 1–13 (2008).
3. Palandri, J. & Kharaka, Y. *A Compilation of Rate Parameters of Water-Mineral Interaction Kinetics for Application to Geochemical Modeling. U.S. Geological Survey Open File Report 2004-1068* (2004).
4. Shock, E. L., Sassani, D. C., Willis, M. & Sverjensky, D. A. Inorganic species in geologic fluids: Correlations among standard molal thermodynamic properties of aqueous ions and hydroxide complexes. *Geochim Cosmochim Acta* **61**, 907–950 (1997).
